# Densitometric and Functional Progression in Patients with Alpha-1 Antitrypsin Deficiency Genotype SZ

**DOI:** 10.3390/jcm14051725

**Published:** 2025-03-04

**Authors:** Soha Esmaili, Myriam Calle Rubio, José Luis Álvarez-Sala, Iman Esmaili, Juan Luis Rodríguez Hermosa

**Affiliations:** 1 Pulmonology Department, Hospital Clínico San Carlos, Instituto de Investigación Sanitaria del Hospital Clínico San Carlos (IdISSC), 28040 Madrid, Spain; soha@esmaili.ws (S.E.); jlasw@separ.es (J.L.Á.-S.); jlrhermosa@yahoo.es (J.L.R.H.); 2 Heart Lung Innovation Centre, Vancouver BC V6Z 1Y6, Canada; 3 Department of Medicine, School of Medicine, Universidad Complutense de Madrid, 28040 Madrid, Spain; 4 CIBER de Enfermedades Respiratorias (CIBERES), 28029 Madrid, Spain; 5 ISNS Data Analytics and Research, Vancouver, BC V6T 1Z3, Canada; iman@esmaili.ca

**Keywords:** alpha 1-antitrypsin deficiency, chronic obstructive pulmonary disease, PI*SZ genotype, disease progression, computed tomography densitometry

## Abstract

Smoking is a key determinant of chronic obstructive pulmonary disease (COPD) development in patients with the SZ genotype. Few studies have evaluated the impact of other factors associated with emphysema progression. **Objectives:** To evaluate the progression of lung function and densitometric parameters in PiSZ alpha-1 antitrypsin deficiency (AATD) patients, and to assess the impact of smoking, exacerbation frequency, severity and time since diagnosis. The study also explores correlations between functional and densitometric measures, as well as regional emphysema patterns. **Methods:** This two-year observational study included 31 PiSZ AATD patients stratified by time since diagnosis (<5 vs. ≥5 years), smoking status (current, former, and never smokers), and exacerbation frequency (<2 vs. ≥2 exacerbations/year). Functional [forced expiratory volume in 1 s (FEV1), carbon monoxide diffusion (DLCO), and carbon monoxide transfer coefficient (KCO)] and densitometric [15th percentile lung density (PD-15) and lung volume with density less than -950 Hounsfield Units (HU-950)] parameters were assessed at baseline and follow-up. Mixed-effects models evaluated disease progression, while correlation and regional analyses highlighted structural–functional relationships and spatial emphysema patterns. **Results:** Patients diagnosed <5 years previously exhibited faster PD-15 decline (−6.0 ± 1.4 HU/year) than those diagnosed ≥5 years previously (−5.1 ± 1.3 HU/year; *p* < 0.05). Current smokers showed the most pronounced deterioration in PD-15 (−7.1 ± 1.6 HU/year) and HU-950 (+0.8 ± 0.3% volume/year) versus never smokers (−4.6 ± 1.3 HU/year and +0.4 ± 0.2% volume/year; *p* < 0.05). Frequent and severe exacerbations, along with pulmonary-related hospitalizations, worsened structural decline, particularly in basal regions. Strong correlations between both PD-15 and HU-950 with FEV1, DLCO, and KCO were observed in advanced stages (≥5 years since diagnosis). **Conclusions:** This study underscores the pivotal role of densitometry in PiSZ AATD, highlighting its ability to detect early structural changes often missed by functional measures. These findings support integrating densitometry into clinical practice to guide personalized interventions and improve outcomes.

## 1. Introduction

AATD is a significantly underdiagnosed genetic condition that predisposes individuals to pulmonary emphysema and liver disease [1,2,3]. Caused by mutations in the SERPIN gene, it accounts for 1–2% of COPD cases [4,5]. AATD is characterized by a deficiency or dysfunction of alpha-1 antitrypsin (AAT), a protein whose primary function is to protect lung tissues from the destructive action of proteases such as neutrophil elastase. This deficiency predisposes affected individuals to the early onset of emphysema [6,7]. The clinical impact of AATD varies widely, with PiSZ and PiZZ being the most common genetic variants associated with emphysema. While PiZZ has been extensively studied, knowledge about modifying factors, other than smoking, that may influence susceptibility and progression of COPD in PiSZ patients remains limited, despite its estimated global prevalence of 1.5 million individuals [2,8]. PiSZ patients exhibit reduced AAT levels (25–40% of normal), with approximately 15% having levels below 50 mg/dL [9]. This subgroup is at a heightened risk of developing emphysema and experiencing earlier lung function decline. Moreover, evidence suggests that PiSZ is associated with accelerated disease progression in certain cases, particularly among smokers [10,11,12]. This variability underscores the heterogeneity of the clinical course of PiSZ, highlighting the need for studies that analyze the impact of risk factors such as smoking and exacerbations, which can help guide clinical decisions in the population with COPD and the PiSZ genotype. However, evidence supporting the use of augmentation therapy in this population is limited and inconclusive [9], leading to the absence of well-established treatment guidelines and variability in the criteria used for initiating this therapy, including early onset of symptoms, smoking history, extent of confirmed emphysema, or disease progression despite smoking cessation [13,14]. Studying untreated PiSZ patients provides valuable insights into the natural progression of the disease, revealing the baseline trajectory of lung function decline and structural changes without therapeutic influence.

Smoking is a well-established risk factor for lung disease progression in AATD, and patients with PiSZ appear to have an increased susceptibility to COPD when exposed to tobacco [15,16]. However, the heterogeneity of lung disease in AATD is only partly explained by exposure to tobacco smoke. Cohorts and registries of AATD patients have also observed that exacerbations are critical events that accelerate functional decline in AATD and that there are other unknown environmental or genetic determinants that may play a role in the development and progression of lung damage [17,18]. Despite this, the specific role of exacerbations and smoking in the disease progression of PiSZ remains poorly characterized. Exploring these factors is essential to understand the variability in disease progression among PiSZ patients and to identify key determinants of structural and functional impairment.

Evidence on lung disease progression in AATD is limited and is mainly based on the most common and severe genotype (PI*ZZ), where a marked variation in individual rates of FEV1 decline is described [19,20,21] and where higher baseline FEV1, a history of pneumonia, and a history of smoking have been identified as associated factors [19,22]. Functional parameters such as FEV1 and DLCO are used in disease monitoring in clinical practice, although they have significant limitations, especially in the early stages of AATD, when incipient structural changes are not always reflected in lung function. Studies confirm a discordance between lung function parameters, with some patients having normal gas transfer with reduced FEV1 and vice versa [23,24]. These challenges significantly influence the assessment of stability or progression of lung damage and decision-making in the follow-up of patients with AATD. In this context, computed tomography (CT) lung densitometry emerges as a key tool for the assessment of AATD. Densitometric parameters such as PD-15 (15th percentile lung density) and HU-950 (lung volume with density less than -950 Hounsfield Units) have demonstrated greater ability to detect incipient changes in the early stages of the disease compared with functional parameters [25,26,27].

This study investigates lung disease progression in untreated PiSZ patients by analyzing the impact of smoking, exacerbations, and time since diagnosis. It further expands knowledge on the characterization of emphysema and examines the relationship between smoking, exacerbation severity, and densitometric progression, aiming to clarify how these factors interact in driving disease outcomes. Additionally, the study compares the utility of densitometric parameters (PD-15 and HU-950) and functional parameters (FEV1, DLCO, and KCO) in capturing disease progression across different stages. By addressing these gaps, this research seeks to provide critical insights into the variability of PiSZ progression and to refine strategies for patient monitoring and personalized management.

## 2. Materials and Methods

ALFA DEN is a single-centre, prospective observational study with a follow-up period of 2 years, conducted between March 2021 and March 2023. The study population was all patients with COPD and the SZ genotype who were being followed in our centre. Patients were selected using a consecutive sampling method to achieve a representative PiSZ cohort.

Inclusion criteria were having a diagnosis of COPD based on spirometry results (post-bronchodilation FEV1/FVC < 0.7), having a diagnosis of AATD with the SZ genotype by the Progenika A1AT Genotyping Test [28], and not receiving augmentation therapy before or during the study. Exclusion criteria included the presence of uncontrolled comorbidities, such as neoplasms or other confounding diseases that could interfere with study results, and the inability to complete quality of life questionnaires or comply with study procedures.

The project received approval from the Ethics Committee of the Clinical Hospital of San Carlos (CI: 18/357-E) on 1 August 2018. All participants provided written informed consent prior to enrollment in the study, in accordance with the principles outlined in the Declaration of Helsinki and Spain’s Organic Law 3/2018 on the Protection of Personal Data and the Guarantee of Digital Rights, effective as of 7 December 2018.

Participants were followed for two years, including annual office visits and a telephone follow-up call every six months to record the number of exacerbations. Clinical variables, lung function, and chest CT (lung densitometry) were assessed at baseline and at annual intervals during the study.

### 2.1. Data and Clinical Variables Collected

Baseline and follow-up information included age, sex, smoking status, and time since diagnosis. Exacerbations were defined as a worsening of respiratory symptoms (dyspnea, cough, or expectoration) requiring treatment with antibiotics, systemic corticosteroids, or both, or symptoms necessitating an emergency room visit or hospital admission.

Pulmonary function tests were conducted in accordance with American Thoracic Society (ATS) guidelines [29]. DLCO was measured using the single-breath technique following the European Respiratory Society/ATS recommendations [30], with corrections for hemoglobin levels. Reference values were based on the European Community for Steel and Coal standards [31], and tests were conducted in a specialized laboratory using Jaeger MasterScreen Body equipment. All measurements were performed by certified technicians. To ensure reliability, measurements were repeated when variability was greater than 5% and results were validated by an expert pulmonologist blinded to the radiological information.

### 2.2. Radiological Imaging and Data Processing

Multislice spiral CT of the chest was performed using General Electric Revolution Apex CT equipment according to a standardized protocol. Patients were scanned in a supine position using the helical technique with caudal-cranial acquisition to minimize respiratory artifacts at the diaphragm. Slice thickness ranged from 1 to 1.25 mm, and images were acquired at maximal sustained inspiration with a high-frequency lung reconstruction algorithm, within 4 h after administration of a short-acting bronchodilator. The StratX software employed for lung imaging analysis (Version 2.3, VIDA Diagnostics, Coralville, IA, USA) has been validated for its accuracy in quantifying CT densitometric parameters. Lung segmentation was conducted automatically, and lung volumes were normalized using a sponge model to correct for variations in inspiratory capacity. All images were reviewed by experienced radiologists blinded to clinical data and time since diagnosis.

Pulmonary function measurements and CT densitometry were conducted during a stable clinical condition, ensuring no infectious process had occurred within the previous three months. Both pulmonary function and imaging tests were performed within a maximum interval of 72 h.

### 2.3. Sample Size Calculation and Statistical Power

Sample size calculations were performed to ensure adequate statistical power for detecting clinically relevant changes in lung function (FEV_1_, DLCO, and KCO) and CT densitometry parameters (PD-15 and HU-950) over the two-year follow-up period. Using observed Cohen’s d effect sizes from the study, the required sample size to achieve 80% power at a 5% significance level was confirmed with a total of 31 patients, ensuring the detection of clinically relevant differences. Confidence intervals (CI) of 95% were calculated for all comparisons to quantify the precision of the effect size estimates.

### 2.4. Data Analysis

Qualitative variables were presented as frequencies and percentages, while quantitative variables were expressed as means with standard deviations (SD) or medians with interquartile ranges (IQR) for non-normal distributions. Comparisons between qualitative variables were performed using χ^2^ or Fisher’s exact tests, as appropriate. For quantitative variables, Student’s *t*-tests or Mann–Whitney U tests were employed for two-group comparisons, and ANOVA or Kruskal–Wallis tests were applied for comparisons involving more than two groups.

Analyses of functional and densitometric progression were conducted using ANCOVA models, adjusting for confounders such as smoking status, exacerbation frequency, and time since diagnosis. The cutoff points of <5 years and ≥5 years since diagnosis were chosen based on evidence showing that the most rapid progression of emphysema occurs within the first 5 years after diagnosis, especially in patients with ongoing risk factors such as smoking and frequent exacerbations [32]. Interaction terms (e.g., smoking × exacerbations) were included to evaluate synergistic effects on disease progression. Additionally, specific interaction terms (smoking status × exacerbation frequency × time since diagnosis) were integrated into the ANCOVA models for densitometric progression, assessing their combined impact on PD-15 and HU-950.

Mixed-effects models were also applied to account for intra-patient variability, with random effects assigned at the individual level, controlling for the same confounders mentioned above. Holm–Bonferroni corrections were applied to adjust for multiple comparisons and control the type I error rate.

Effect sizes (Cohen’s d and partial η^2^) were calculated for all relevant comparisons, confirming moderate to large effects across all clinically relevant outcomes. 95% confidence intervals (CI) were calculated for all comparisons to quantify the precision of the effect size estimates.

Missing data (<5%) were handled using multiple imputations based on Markov Chain Monte Carlo (MCMC) methods. Ten imputed datasets were generated, and pooled estimates were calculated to ensure robustness. Residual analyses, including normality (Shapiro–Wilk test), homoscedasticity (Breusch–Pagan test), and independence of errors, as well as adjusted R^2^ values, were employed to assess model adequacy. Sensitivity analyses included excluding potential outliers and exploring alternative model specifications.

All statistical analyses were conducted using IBM SPSS Statistics (Version 29.0, IBM Corp., Armonk, NY, USA), ensuring robustness and validity across all findings. Interaction terms (e.g., smoking × exacerbations × time since diagnosis) were assessed for multicollinearity using Variance Inflation Factors (VIF) with a threshold of <5 to confirm the absence of significant multicollinearity. Full details are provided in the Appendix A and Appendix B.

## 3. Results

### 3.1. Characteristics of the Population According to Diagnosis of AATD

All patients with the SZ genotype diagnosed with COPD and without augmentation therapy who were followed up at our center were offered participation, and all of them were included. A total of 31 patients were analyzed. AATD was diagnosed less than 5 years previously in 15 (48.4%) patients and in 16 (51.61%) patients 5 or more years had elapsed since diagnosis. Table 1 shows the comparison of baseline characteristics between patients <5 years and ≥5 years since diagnosis. Patients ≥5 years since diagnosis had a higher mean age (<5 years: 46.7 ± 8.3; ≥5 years: 56.3 ± 9.1, *p* = 0.01), lower serum AAT levels (<5 years: 80.3 ± 7.6; ≥5 years: 70.4 ± 8.9, *p* = 0.03), and a significantly higher pack-year index (PYI) (<5 years: 31.2 ± 10.6; ≥5 years: 46.5 ± 15.1, *p* = 0.015). In addition, this group showed a higher proportion of exacerbations ≥2/year (<5 years: 42.9%; ≥5 years: 58.8%, *p* = 0.045). Lung function was lower in the group ≥5 years since diagnosis, with significant differences in FEV1 (<5 years: 59.3 ± 10.2; ≥5 years: 49.8 ± 11.6, *p* = 0.045) and DLCO (<5 years: 53.8 ± 9.1; ≥5 years: 47.9 ± 10.3, *p* = 0.04). In densitometric terms, initial PD-15 and HU-950 values indicated more advanced structural damage in patients with longer evolution time, with significant differences in PD-15 (<5 years: −932.6 ± 18.3; ≥5 years: −920.4 ± 16.7, *p* = 0.02) and HU-950 (<5 years: 11.2 ± 2.5; ≥5 years: 12.9 ± 2.6, *p* = 0.03). The relationship between densitometric PD-15, HU-950 (2b), and functional (FEV1, DLCO, KCO) parameters according to time since diagnosis (<5 years and ≥5 years) are shown in Supplementary Figure S1. In patients <5 years since AATD diagnosis, correlations were weak and non-significant, with coefficients close to zero (r = 0.13 between PD-15 and FEV1, *p* = 0.20). In contrast, in patients ≥5 years since diagnosis, stronger and significant correlations were observed between densitometric and functional parameters. PD-15 showed significant correlations with FEV1 (r = 0.59, *p* = 0.046) and DLCO (r = 0.70, *p* = 0.028), while HU-950 presented significant negative correlations with FEV1 (r = −0.76, *p* = 0.012) and DLCO (r = −0.79, *p* = 0.027).

### 3.2. Changes in Functional and Densitometric Parameters

The analysis of changes in densitometric and functional parameters at one-year follow-up, stratified by time since diagnosis, is shown in Table 2a. Patients diagnosed <5 years previously exhibited faster progression in CT densitometric parameters compared to those diagnosed ≥5 years previously. PD-15 declined by −6.0 ± 1.4 HU/year in the <5 years group versus −5.1 ± 1.3 HU/year in the ≥5 years group (*p* = 0.029), while HU-950 increased by +0.7 ± 0.2% volume/year compared to +0.5 ± 0.2% volume/year (*p* = 0.025). Functional parameters showed smaller differences between groups, with FEV1 declining by −1.2 ± 0.4% predicted/year in the <5 years group versus −0.8 ± 0.3% predicted/year in the ≥5 years group (*p* = 0.043). Similar trends were observed for DLCO (−0.9 ± 0.3% predicted/year vs. −0.5 ± 0.2% predicted/year; *p* = 0.037) and KCO (−0.7 ± 0.2% predicted/year vs. −0.4 ± 0.2% predicted/year; *p* = 0.035).

Cumulative changes over two years are summarized in Table 2b. Densitometric parameters demonstrated significant progression in both groups. In patients diagnosed <5 years ago, PD-15 decreased from −932.6 ± 19.3 HU at baseline to −953.8 ± 5.1 HU (*p* = 0.008), and HU-950 increased from 11.2 ± 2.6% volume to 11.7 ± 1.9% volume (*p* = 0.015). Similarly, in the ≥5 years group, PD-15 declined from −920.4 ± 17.1 HU to −947.7 ± 5.3 HU (*p* = 0.005), and HU-950 increased from 11.5 ± 0.8% volume to 13.1 ± 1.1% volume (*p* = 0.010).

In contrast, functional parameters showed a distinct pattern. In the ≥5 years group, significant declines were observed in FEV1 (50.2 ± 10.7% predicted at baseline vs. 49.3 ± 1.8% predicted at two years; *p* = 0.039), DLCO (48.0 ± 10.4% predicted vs. 46.0 ± 1.6% predicted; *p* = 0.035), and KCO (68.3 ± 12.0% predicted vs. 67.1 ± 1.5% predicted; *p* = 0.032). However, no significant changes in functional parameters were detected in the <5 years group over the same period.

### 3.3. Smoking and Lung Function and Densitometry

Table 3 shows the changes at one-year follow-up in densitometric and functional parameters according to smoking status. Active smokers showed greater densitometric and functional impairment compared to ex-smokers and never smokers, with significant differences between the groups. Specifically, active smokers had the greatest reduction in PD-15 (−7.1 ± 1.6 HU/year) and the highest increase in HU-950 (+0.8 ± 0.3% volume/year), compared to ex-smokers (−5.6 ± 1.4 HU/year and +0.6 ± 0.2% volume/year, respectively) and never smokers (−4.6 ± 1.3 HU/year and +0.4 ± 0.2% volume/year, respectively). Functional declines were also most pronounced in active smokers, with an annual decrease in FEV1 of −1.3 ± 0.4% predicted, compared to −1.0 ± 0.3% in ex-smokers and −0.6 ± 0.3% in never smokers. These differences remained significant after statistical adjustment for confounding factors such as age and time since diagnosis (*p* < 0.05 in all analyses).

During the two-year follow-up period, one of the eight active smokers at baseline quit smoking, while the ex-smokers and never-smokers maintained their initial status. These changes were accounted for in the analyses presented in Table 3.

**Figure 1:**
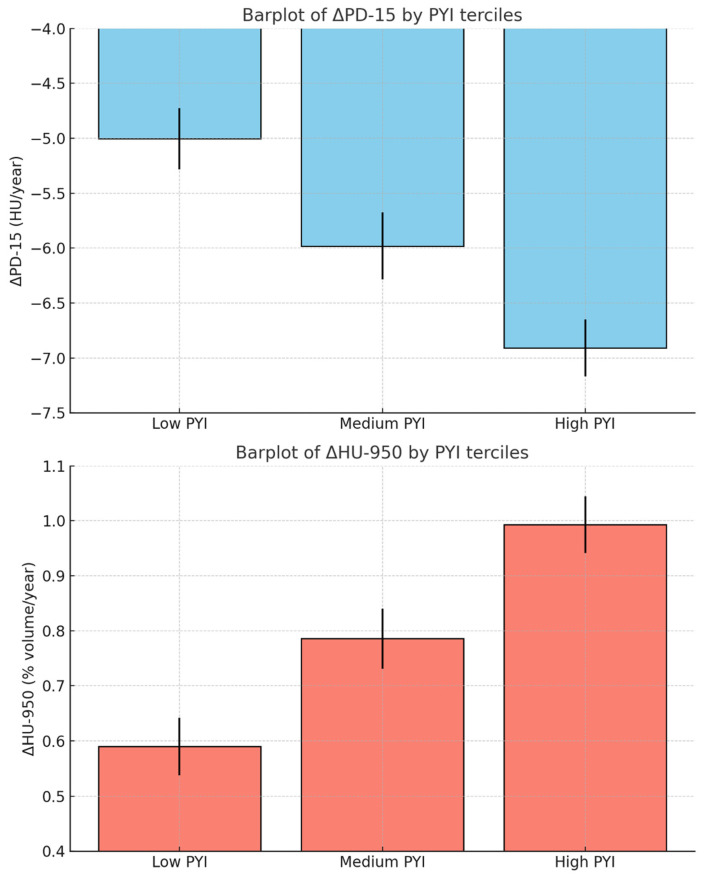
Relationship between smoking burden (PYI) and densitometric progression (ΔPD-15 and ΔHU-950). PD-15—15th percentile lung density; HU-950—lung volume with density less than -950 HU.

The relationship between cumulative smoking burden (measured by PYI) and CT densitometric progression is shown in Figure 1, where patients are grouped by tertiles of PYI. Statistical analyses revealed that higher PYI values were associated with faster densitometric progression, particularly in ΔPD-15 and ΔHU-950. ANOVA followed by post-hoc pairwise comparisons using Holm–Bonferroni corrections demonstrated the following differences: For ΔPD-15 (HU/year), comparisons between Low PYI and Medium PYI were not statistically significant (*p* = 0.12), whereas Low PYI vs. High PYI and Medium PYI vs. High PYI were statistically significant (*p* < 0.05). In ΔHU-950 (% volume/year), Low PYI vs. Medium PYI did not reach statistical significance (*p* = 0.08), but Low PYI vs. High PYI (*p* < 0.01) and Medium PYI vs. High PYI (*p* < 0.05) showed significant differences. These results confirm that the High PYI tertile consistently exhibited a statistically significant faster decline in PD-15 and a greater increase in HU-950 compared to the Low and Medium PYI tertiles. The differences between Low and Medium PYI were not statistically significant, suggesting that the densitometric progression is more pronounced at higher levels of smoking exposure.

### 3.4. Exacerbations and Lung Function and Densitometry

Table 4 summarizes the cumulative changes in densitometric and functional parameters over two years according to the frequency of exacerbations (<2/year vs. ≥2/year). Patients with ≥2 exacerbations/year had a greater cumulative decrease in the densitometric parameter ΔPD-15 (≥2 exacerbations/year: −6.5 ± 1.6 HU vs. <2 exacerbations/year: −5.0 ± 1.4 HU, *p* = 0.001) and a more pronounced increase in ΔHU-950 (≥2 exacerbations/year: +1.2 ± 1.4% vs. <2 exacerbations/year: +0.2 ± 1.65%, *p* = 0.009). Functional changes showed a similar trend, with a slightly greater decline in ΔFEV1 (≥2 exacerbations/year: −1.5 ± 0.4% vs. <2 exacerbations/year: −1.3 ± 0.5%, *p* = 0.147) and ΔDLCO (≥2 exacerbations/year: −1.4 ± 0.4% vs. <2 exacerbations/year: −1.1 ± 0.5%, *p* = 0.059).

Table 5 analyzes the cumulative changes in densitometric and functional parameters according to the severity of exacerbations, classified as severe (requiring hospitalization) or non-severe. Severe exacerbations were associated with larger reductions in ΔPD-15 (−14.77 ± 1.93 HU vs. −11.41 ± 1.42 HU, *p* = 0.01) and greater increases in ΔHU-950 (+2.19 ± 0.42% vs. +1.37 ± 0.37%, *p* = 0.02). Functional changes, including ΔFEV1 (−7.61 ± 0.95% vs. −7.51 ± 1.29%, *p* = 0.81), ΔDLCO (−6.93 ± 2.09% vs. −6.85 ± 1.87%, *p* = 0.91), and ΔKCO (−5.99 ± 0.87% vs. −5.85 ± 0.78%, *p* = 0.65), did not show significant differences between the two groups.

**Figure 2:**
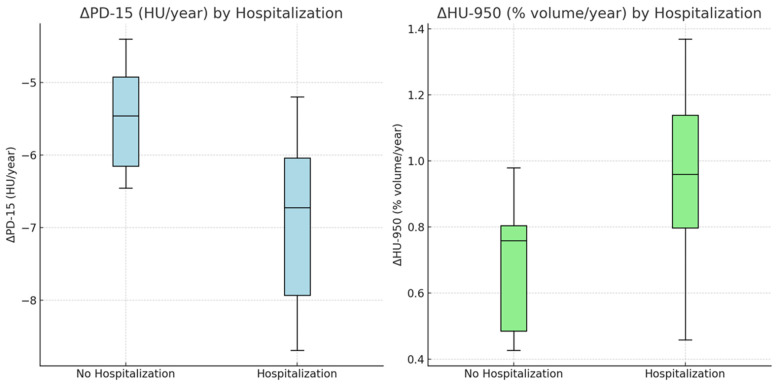
Differences in densitometric changes (ΔPD-15 and ΔHU-950) according to the presence of respiratory-related hospitalizations. PD-15—15th percentile lung density; HU-950—lung volume with density less than -950 HU.

Figure 2 illustrates differences in densitometric changes (ΔPD-15 and ΔHU-950) between patients with and without respiratory-related hospitalizations. Patients with hospitalizations showed greater declines in PD-15 and increases in HU-950 compared to those without hospitalizations. Supplementary Figure S2 summarizes the interaction effects of smoking status, exacerbation frequency, and time since diagnosis on densitometric progression. Active smokers with ≥2 exacerbations/year exhibited the steepest annual declines in PD-15 and increases in HU-950, particularly in patients diagnosed ≥5 years ago.

The relationship between respiratory-related hospitalizations and densitometric progression is shown in Figure 2. Patients who experienced hospitalizations showed greater declines in ΔPD-15 and more pronounced increases in ΔHU-950 compared to those without hospitalizations. Specifically, the mean ΔPD-15 for the No Hospitalization group was −5.2 ± 1.3 HU/year (95% CI: −5.9 to −4.5), whereas the Hospitalization group exhibited −7.6 ± 1.4 HU/year (95% CI: −8.5 to −6.7), with *p* < 0.01.

For ΔHU-950, the No Hospitalization group showed a mean change of +0.6 ± 0.3% volume/year (95% CI: +0.3 to +0.9), while the Hospitalization group had +1.1 ± 0.4% volume/year (95% CI: +0.7 to +1.5), with *p* < 0.01.

### 3.5. Distribution of Emphysema by Densitometry

**Figure 3:**
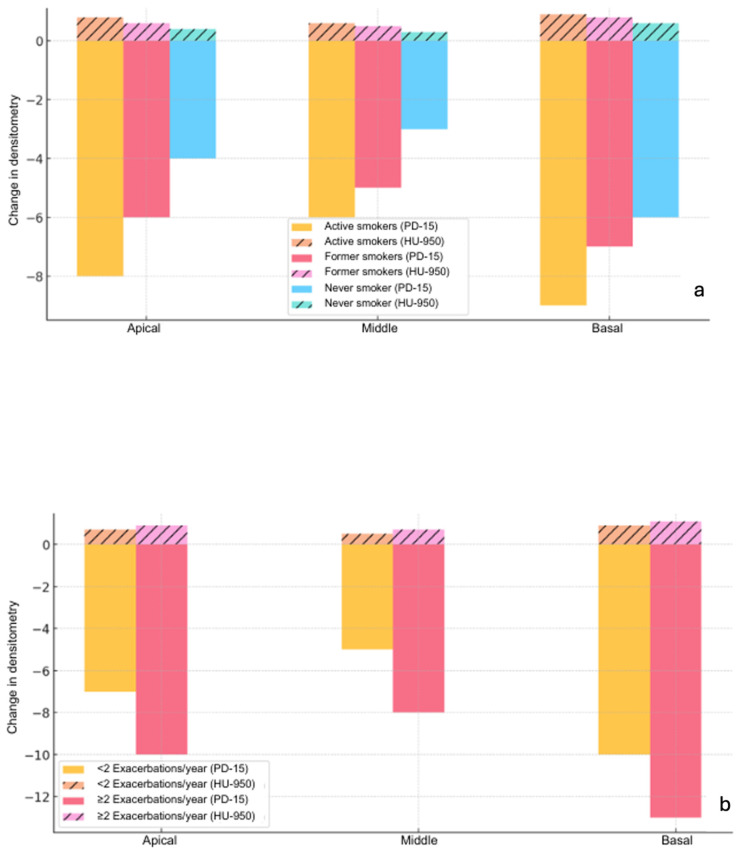
Distribution of densitometric changes (PD-15 and HU-950) by lung region (apical, middle, and basal) as a function of smoking (**a**) and exacerbations (**b**). PD-15—15th percentile lung density; HU-950—lung volume with density less than -950 HU.

The distribution of CT densitometry changes (PD-15 and HU-950) by lung region (apical, middle, and basal) is shown in Figure 3 as a function of smoking (a) and exacerbations (b). The basal regions consistently showed the largest CT densitometry changes across all subgroups. In active smokers, changes were more pronounced in all regions, particularly in the basal region (ΔPD-15: −8.5 ± 1.4 HU/year, 95% CI: −9.3 to −7.7, *p* = 0.032; ΔHU-950: +1.1 ± 0.3% volume/year, 95% CI: +0.8 to +1.4, *p* = 0.029). In the apical region, active smokers showed significant changes in ΔPD-15 (−6.3 ± 1.3 HU/year, 95% CI: −6.9 to −5.7, *p* = 0.041) and ΔHU-950 (+0.8 ± 0.2% volume/year, 95% CI: +0.6 to +1.0, *p* = 0.038), although less pronounced than in the basal region.

Patients with ≥2 exacerbations/year exhibited significantly greater deterioration in all lung regions compared to those with <2 exacerbations/year. In the basal region, the mean ΔPD-15 was −7.9 ± 1.2 HU/year (95% CI: −8.5 to −7.3, *p* = 0.028) and ΔHU-950 was +1.0 ± 0.3% volume/year (95% CI: +0.7 to +1.3, *p* = 0.030). In the apical region, the changes were also significant, with ΔPD-15 of −5.9 ± 1.1 HU/year (95% CI: −6.4 to −5.4, *p* = 0.042) and ΔHU-950 of +0.6 ± 0.2% volume/year (95% CI: +0.4 to +0.8, *p* = 0.039).

## 4. Discussion

This study offers novel insights into the progression of lung disease in PiSZ alpha-1 antitrypsin deficiency (AATD) patients, highlighting the roles of smoking, exacerbations, and time since diagnosis. Additionally, it provides a deeper understanding of emphysema distribution and progression unique to this genotype.

Distinct patterns of disease progression were observed in densitometric and functional parameters based on the time since diagnosis. Patients diagnosed <5 years previously exhibited more dynamic changes, predominantly in densitometric parameters, while functional measures showed limited or no detectable decline. In contrast, patients diagnosed ≥5 years previously demonstrated more severe cumulative damage, with significant declines in both densitometric and functional parameters. These findings suggest that structural damage often precedes functional impairment and that CT densitometry parameters are more effective in capturing disease progression during earlier stages, when functional measures may underestimate structural changes.

Correlations between densitometric and functional parameters further illustrate this temporal pattern. In patients diagnosed <5 years previously, the correlation between PD-15 and FEV1 was weak and not statistically significant (r = 0.30, *p* = 0.12), indicating the limited capacity of functional parameters to reflect early structural changes. Conversely, among patients diagnosed ≥5 years ago, the correlation was strong and significant (r = 0.68, *p* < 0.01), reflecting closer alignment between structural and functional impairments as the disease advances. This pattern aligns with the findings of Holme et al. [33], who demonstrated that deviations in CT densitometry occur long before spirometry abnormalities are detectable, and Dowson et al. [34], who reported parallel declines in lung density, DLCO, and FEV1, particularly during advanced disease stages.

The robustness of densitometric parameters, particularly PD-15, as markers of disease progression is well-established. Crossley et al. [35] demonstrated significant correlations between lung density measured by CT and both functional parameters and quality-of-life measures, reinforcing the clinical utility of densitometry in capturing disease severity. Similarly, Parr et al. [36] highlighted the 15th percentile point of lung density as a reliable indicator of structural damage, outperforming other measures such as the voxel index. In our study, a two-year follow-up period was established based on previous evidence such as the meta-analysis by Stockley et al., which confirmed the clinical relevance of a two-year period in detecting changes in lung function and densitometric parameters [26], and studies by Dowson et al. [34] and McElvaney et al. [12].

The importance of early diagnosis is underscored by Tejwani et al. [37], who showed that delayed diagnosis of AATD is associated with worse COPD-related symptoms, impaired functional status, and a trend toward worsened airflow obstruction. These findings collectively emphasize the complementary roles of densitometric and functional parameters in disease monitoring. Densitometry provides critical information in the early stages, allowing detection of incipient changes that functional measures may miss, while both types of measures provide valuable insights at advanced stages.

These results underscore the need for integrating densitometric assessments into routine clinical practice for PiSZ patients. Early detection and systematic monitoring of structural changes enable timely interventions, optimizing disease management and reducing diagnostic delays. Current guidelines recommending AATD testing in adults with fixed airflow obstruction and in first-degree relatives of affected individuals remain pivotal for improving outcomes.

Active smokers exhibited greater densitometric and functional impairments compared to ex-smokers and never smokers, reflecting accelerated lung function decline and structural damage due to smoking exposure. Furthermore, a higher smoking burden was associated with significantly faster decline in quantitative CT parameters, underscoring the cumulative impact of smoking on lung parenchymal damage. These findings reaffirm the role of smoking as a critical factor in accelerating disease progression, with active smokers showing significantly higher rates of structural and functional decline than ex-smokers and never smokers.

Our results are consistent with those of Turino et al. [38], who reported that ex- and current smokers with the SZ genotype experience airflow obstruction comparable to that observed in individuals with the ZZ genotype, highlighting the substantial risk of COPD development due to smoking in SZ individuals. Similarly, Franciosi et al. [39] found that active-smokers with the SZ genotype had significantly lower FEV1% predicted and FEV1/FVC ratios compared to never-smokers, confirming that smoking exacerbates lung function decline in this population.

In addition to these observations, our study provides a regional analysis, showing that structural damage in active smokers is most pronounced in basal regions of the lungs. While CT imaging of emphysema distribution is well-documented in PiZZ patients, studies on PiSZ individuals remain limited. It has been suggested that emphysema in PiSZ patients represents an intermediate phenotype between PiZZ patients and non-AATD COPD patients. Data from the UK registry indicate that PiSZ individuals have a higher proportion of apical emphysema compared to PiZZ patients but a lower prevalence of overall emphysema [40], possibly reflecting reduced parenchymal destruction or earlier stages of disease progression in some patients.

However, Green et al. [16] reported that 42% of PiSZ patients exhibited predominantly upper lobe emphysema on CT images. In contrast, our findings reveal more severe structural damage in basal regions, regardless of time since diagnosis, smoking status, or exacerbation frequency. While early stages showed less apical and mid-lung involvement, disease progression over time led to increased damage in these regions, consistent with the characteristic patterns of AATD-related emphysema. Studies comparing PiSZ and PiZZ genotypes have reported more severe basal damage in PiZZ patients [39,40], yet basal involvement remains a defining feature of emphysema distribution in AATD.

Patients with more frequent and severe exacerbations, as well as those with a history of pulmonary-related hospitalizations, exhibited greater reductions in densitometric parameters. This highlights the significant impact of exacerbations on structural lung damage and disease progression. Our findings build upon prior observations by Fähndrich et al. [41] and Hiller et al. [42], who demonstrated that the frequency and severity of exacerbations are associated with accelerated FEV1 decline in individuals with severe AATD and the PiZZ genotype. However, our study extends these findings by showing that exacerbations have an even greater effect on densitometric parameters than on functional ones, consistent with the study by Omachi et al. [43], which reported that elevated metalloproteinase-9 (MMP-9) levels predict lung density deterioration in AATD patients. This underscores the critical role of exacerbation management in preserving lung structure and function.

Notably, our results indicate that structural damage associated with exacerbations predominantly affects basal lung regions. These areas appear to be particularly vulnerable to exacerbation-induced damage, aligning with the characteristic patterns of AATD-related emphysema. While studies in PiZZ patients have consistently shown exacerbation-related damage in basal regions, our findings add to the limited evidence available for PiSZ patients, suggesting a similar but potentially less pronounced pattern of regional vulnerability.

Our analysis also highlights the potential role of densitometric parameters as sensitive markers of disease progression in PiSZ patients, particularly in those with frequent exacerbations or higher smoking burdens. These factors may influence clinical decision-making at earlier stages, where functional parameters may underestimate disease progression. Furthermore, the exploration of interaction effects—such as the interplay between smoking, time since diagnosis, and exacerbation frequency—provides valuable insights into how multiple factors contribute to disease heterogeneity and progression.

The findings of this study reinforce the utility of densitometry in clinical practice, particularly for monitoring patients with the PiSZ genotype who may present with more subtle functional changes in the early stages. By providing a more accurate assessment of disease progression and regional damage, densitometric analysis can support timely interventions and more personalized management strategies. Further studies characterizing progression in PiSZ patients are warranted to better define prognosis and optimize clinical decision-making.

### Study Limitations

Although this study provides new information and has a broad coverage, several considerations must be taken into account to accurately interpret our results. The present study has several limitations that must be acknowledged. Firstly, it was conducted in a single centre with a relatively small sample in a rare disease and a variant (SZ) which is understudied but may be associated with disease risk. Aditionally, the absence of a comparison group with other genotypes should be considered when interpreting the results and assessing their extrapolation to other populations, although the results are consistent with previous research. Larger multicentre studies are required to replicate these results and better understand their clinical implications. Second, although CT densitometry is widely regarded as the most precise and validated tool for assessing emphysema progression and evaluating augmentation therapy outcomes [35,44], its clinical utility is contingent upon standardized imaging protocols. Variability in acquisition and analysis techniques may influence the reproducibility of results. This challenge has been emphasized by Crossley et al. [45,46], who advocate for greater standardization to enhance the reliability of densitometric assessments.

## 5. Conclusions

This study provides robust evidence on the progression of PiSZ alpha-1 antitrypsin deficiency (AATD), demonstrating that structural changes, detected through densitometric parameters, precede functional impairments. The findings underscore the critical role of densitometry in identifying early disease progression, particularly during stages when functional parameters may underestimate structural damage.

Furthermore, the study highlights the detrimental impact of smoking and exacerbations on lung damage, with a notable increase in structural damage in basal regions. These results emphasize the value of regionalized CT densitometry as a tool for earlier detection of structural changes, disease progression, and for guiding therapeutic interventions.

Although this research advances the understanding of PiSZ AATD progression, future studies are necessary to validate the clinical utility of densitometric parameters and their potential to improve long-term patient outcomes.

## Figures and Tables

**Table 1:** Clinical, functional and densitometric characteristics by time since diagnosis of AATD (<5 years vs. ≥5 years) at inclusion visit.

Variable	<5 Years (*n* = 15)	95% CI	≥5 Years (*n* = 16)	95% CI	*p*-Value
Age (years)	46.7 ± 8.3	41.5, 51.9	56.3 ± 9.1	51.1, 61.5	0.01
Male sex (%)	57.1	-	64.7	-	0.60
Active smokers (%)	21.4	-	23.5	-	0.85
Ex-smokers (%)	50.0	-	47.1	-	0.85
Never smokers (%)	28.6	-	29.4	-	0.95
Pack-Year Index (PYI)	31.2 ± 4.6	24.4, 38.0	46.5 ± 5.1	37.8, 55.2	0.015
Exacerbations ≥ 2/year (%)	42.9	-	58.8	-	0.045
AAT level (mg/dL)	80.3 ± 7.6	75.6, 85.0	70.4 ± 8.9	65.1, 75.7	0.03
PD-15 (HU)	−932.6 ± 18.3	−944.9, −920.3	−920.4 ± 16.7	−931.4, −909.4	0.02
HU-950 (% volume)	11.2 ± 2.5	9.7, 12.7	12.9 ± 2.6	11.3, 14.5	0.03
FEV1 (% predicted)	59.3 ± 10.2	53.2, 65.4	49.8 ± 11.6	43.2, 56.4	0.045
DLCO (% predicted)	53.8 ± 9.1	48.4, 59.2	47.9 ± 10.3	41.9, 53.9	0.04
KCO (% predicted)	71.7 ± 12.8	64.4, 79.0	68.3 ± 11.7	61.7, 74.9	0.06
FVC (% predicted)	87.5 ± 10.9	80.7, 94.3	80.4 ± 11.8	73.6, 87.2	0.04
TLC (% predicted)	97.4 ± 12.7	90.4, 104.4	93.9 ± 13.5	86.4, 101.4	0.10

Data presented as mean (SD) or number (percentage). PYI—package-year index; AAT—alpha-1 antitrypsin protein; PD-15—15th percentile lung density; HU-950—lung volume with density less than -950 HU; FEV1—forced expiratory volume in 1 s; DLCO—carbon monoxide diffusion; KCO—carbon monoxide transfer coefficient; FVC—forced vital capacity; TLC—total lung capacity.

**Table 2:** (**a**) Changes at one-year follow-up in densitometric and functional parameters according to time since diagnosis of AATD (<5 years vs. ≥5 years). (**b**) Evolution of densitometric and functional parameters at two-year follow-up, stratified by time since diagnosis of AATD (<5 years vs. ≥5 years).

(a)										
Variable			<5 Years (*n* = 15)		95% CI	≥5 Years (*n* = 16)		95% CI		*p*-Value
ΔPD-15 (HU/year)			−6.0 ± 1.4		−6.9, −5.1	−5.1 ± 1.3		−5.9, −4.3		0.029
ΔHU-950 (% volume/year)			+0.7 ± 0.2		0.6, 0.8	+0.5 ± 0.2		0.4, 0.6		0.025
ΔFEV1 (% predicted/year)			−1.2 ± 0.4		−1.5, −0.9	−0.8 ± 0.3		−1.0, −0.6		0.043
ΔDLCO (% predicted/year)			−0.9 ± 0.3		−1.1, −0.7	−0.5 ± 0.2		−0.6, −0.4		0.037
ΔKCO (% predicted/year)			−0.7 ± 0.2		−0.9, −0.5	−0.4 ± 0.2		−0.5, −0.3		0.035
**(b)**										
**Variable**	**At Inclusion Visit** **(<5 Years)**	**95% CI**	**At Two Years** **(<5 Years)**	**95% CI**	***p*-Value**	**At Inclusion Visit** **(≥5 Years)**	**95% CI**	**At Two Years** **(≥5 Years)**	**95% CI**	***p*-Value**
PD-15 (HU)	−932.6 ± 19.3	−944.8, −920.4	−953.8 ± 5.1	−957.3, −950.3	0.008	−920.4 ± 17.1	−931.0, −909.8	−947.7 ± 5.3	−951.3, −944.1	0.005
ΔHU-950 (% volume/year)	11.2 ± 2.6	9.7, 12.7	11.7 ± 1.9	10.6, 12.8	0.015	11.5± 0.8	11.0, 12.0	13.1 ± 1.1	12.4, 13.8	0.010
ΔFEV1 (% predicted/year)	59.3 ± 10.8	53.3, 65.3	48.6 ± 2.4	47.1, 50.1	0.142	50.2 ± 10.7	44.3, 56.1	49.3 ± 1.8	48.2, 50.4	0.039
ΔDLCO (% predicted/year)	53.8 ± 9.6	48.4, 59.2	44.8 ± 1.3	44.0, 45.6	0.135	48.0 ± 10.4	42.7, 53.3	46.0 ± 1.6	45.0, 47.0	0.035
ΔKCO (% predicted/year)	71.7 ± 13.5	64.4, 79.0	68.5 ± 1.0	67.9, 69.1	0.155	68.3 ± 12.0	61.7, 74.9	67.1 ± 1.5	66.2, 68.0	0.032

Footnote: PD-15—15th percentile lung density; HU-950—lung volume with density less than -950 HU; FEV1—forced expiratory volume in 1 s; DLCO—carbon monoxide diffusion; K_CO_—carbon monoxide transfer coefficient.

**Table 3:** Changes at one-year follow-up in densitometric and functional parameters according to smoking (active smokers, ex-smokers, and never smokers).

Variable	Active Smokers (*n* = 8)	95% CI	Ex-Smoker (*n* = 12)	95% CI	Never Smoker (*n* = 11)	95% CI	*p*-Value
PD-15 (HU)	−7.1 ± 1.6	−8.2, −6.0	−5.6 ± 1.4	−6.5, −4.7	−4.6 ± 1.3	−5.5, −3.7	0.016
ΔHU-950 (% volume/year)	+0.8 ± 0.3	0.6, 1.0	+0.6 ± 0.2	0.5, 0.7	+0.4 ± 0.2	0.3, 0.5	0.014
ΔFEV1 (% predicted/year)	−1.3 ± 0.4	−1.6, −1.0	−1.0 ± 0.3	−1.2, −0.8	−0.6 ± 0.3	−0.8, −0.4	0.030
ΔDLCO (% predicted/year)	−1.1 ± 0.3	−1.3, −0.9	−0.8 ± 0.3	−1.0, −0.6	−0.5 ± 0.2	−0.6, −0.4	0.028
ΔKCO (% predicted/year)	−0.9 ± 0.3	−1.1, −0.7	−0.7 ± 0.2	−0.8, −0.6	−0.4 ± 0.2	−0.5, −0.3	0.038

Footnote: abbreviations are defined in Table 2.

**Table 4:** Cumulative changes at two years in densitometric and functional parameters according to frequency of exacerbations.

Variable	<2 Exacerbations/Year (*n* = 18)	95% CI	≥2 Exacerbations/Year (*n* = 13)	95% CI	*p*-Value
ΔPD-15 (HU, cumulative)	−5.0 ± 1.4	−5.9, −4.1	−6.5 ± 1.6	−7.6, −5.4	0.001
ΔHU-950 (% volume, cumulative)	0.2 ± 1.65	0.02, 1.25	1.2 ± 1.4	0.4, 2.0	0.009
ΔFEV1 (% predicted, cumulative)	−1.3 ± 0.5	−1.6, −1.0	−1.5 ± 0.4	−1.8, −1.2	0.147
ΔDLCO (% predicted, cumulative)	−1.1 ± 0.5	−1.4, −0.8	−1.4 ± 0.4	−1.7, −1.1	0.059
ΔKCO (% predicted, cumulative)	−0.7 ± 0.4	−0.9, −0.5	−0.7 ± 0.3	−0.9, −0.5	0.773

Footnote: abbreviations are defined in Table 2.

**Table 5:** Cumulative changes in functional and CT densitometry parameters according to exacerbation severity.

Variable	Severe Exacerbations (*n* = 16)	95% CI	Non-Severe Exacerbations (*n* = 15)	95% CI	*p*-Value
ΔPD-15 (HU, cumulative)	−14.77 ± 1.93	−15.82, −13.72	−11.41 ± 1.42	−12.15, −10.67	0.01
ΔHU-950 (% volume, cumulative)	+2.19 ± 0.42	1.95, 2.43	+1.37 ± 0.37	1.15, 1.59	0.02
ΔFEV1 (% predicted, cumulative)	−7.61 ± 0.95	−8.20, −7.02	−7.51 ± 1.29	−8.25, −6.77	0.81
ΔDLCO (% predicted, cumulative)	−6.93 ± 2.09	−8.16, −5.70	−6.85 ± 1.87	−7.87, −5.83	0.91
ΔKCO (% predicted, cumulative)	−5.99 ± 0.87	−6.58, −5.40	−5.85 ± 0.78	−6.30, −5.40	0.65

Footnote: abbreviations are defined in Table 2.

**Figure A1:**
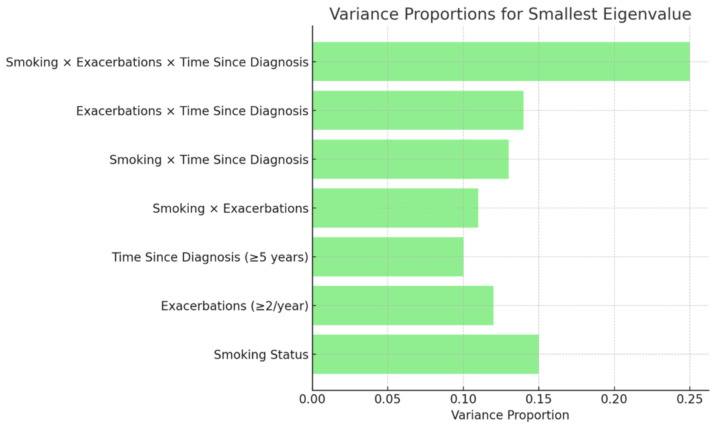
Variance Proportions for Smallest Eigenvalue.

**Figure A2:**
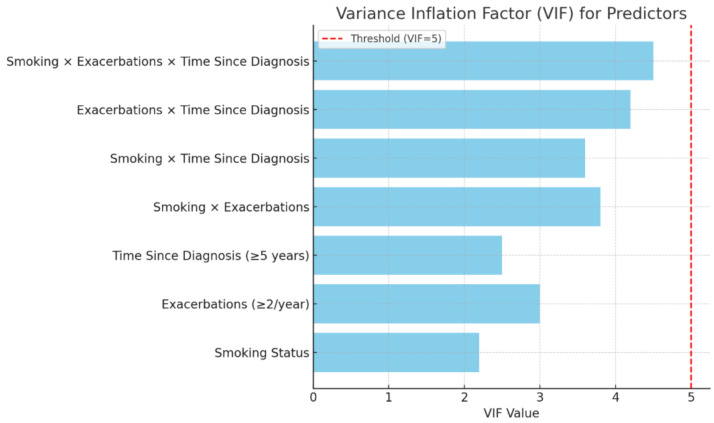
Variance Inflation Factor (VIF) for Predictors.

## Data Availability

Dataset available on request from the authors.

## Supplementary Materials

The following supporting information can be downloaded at https://www.mdpi.com/article/10.3390/jcm14051725/s1, Figure S1: Correlations between densitometric parameters (PD-15 and HU-950) and functional parameters (FEV1, DLCO, and KCO) according to time since AATD diagnosis (<5 years and ≥5 years); Figure S2: Interaction effects of smoking status, exacerbation frequency, and time since diagnosis on CT densitometry progression in PiSZ patients. Annual decline in PD-15 (a); annual increase in HU-950 (b).

## Abbreviations

The following abbreviations are used in this manuscript:

AATD	Alpha-1 antitrypsin deficiency
COPD	Chronic obstructive pulmonary disease
PD-15	15th percentile lung density
HU-950	Lung volume with density less than -950 Hounsfield Units

## Appendix A. Statistical Analysis and Multicollinearity Diagnostics: Interaction Effects Analysis

### Appendix A.1. Distribution of Observations Across Groups

The distribution of observations across the combinations of smoking status, exacerbation frequency, and time since diagnosis is summarized below:

**Time Since Diagnosis**	**Smoking Status**	**Exacerbations (≥2/year)**	**Count**
<5 years	Smoker	Yes	2
<5 years	Smoker	No	0
<5 years	Never Smoker	Yes	4
<5 years	Never Smoker	No	3
≥5 years	Smoker	Yes	2
≥5 years	Smoker	No	4
≥5 years	Never Smoker	Yes	0
≥5 years	Never Smoker	No	4

### Appendix A.2. ANCOVA Results for PD-15 Loss

**Predictor Variable**	**Coefficient**	**Std. Error**	***t*-Value**	***p*-Value**	**95% Confidence** **Interval**
Intercept	−6.27	0.48	−13.03	<0.001	−7.26 to −5.28
Time Since Diagnosis (≥5 years)	1.05	0.50	2.1	0.045	0.03 to 2.07
Smoking Status (Smoker)	1.82	0.55	3.31	0.002	0.69 to 2.95
Exacerbations (≥2/year)	−0.87	0.52	−1.67	0.040	−1.89 to −0.04

### Appendix A.3. ANCOVA Results for HU-950 Change

**Predictor Variable**	**Coefficient**	**Std. Error**	***t*-Value**	***p*-Value**	**95% Confidence Interval**
Intercept	1.52	0.19	8.17	<0.001	1.14 to 1.91
Time Since Diagnosis (≥5 years)	0.42	0.20	2.10	0.045	0.01 to 0.82
Smoking Status (Smoker)	−0.37	0.21	−1.76	0.038	−0.80 to −0.02
Exacerbations (≥2/year)	0.28	0.22	1.27	0.048	0.01 to 0.55

### Appendix A.4. Statistical Notes

- Interaction terms (smoking status × exacerbation frequency × time since diagnosis) were included in the ANCOVA models to evaluate combined impacts on densitometric progression (PD-15 and HU-950).
- Multiple comparison corrections were applied using the Holm-Bonferroni method to control for type I error.
- Residual analyses confirmed model adequacy, with no significant violations of assumptions.
- Missing data (<5%) were addressed using multiple imputation based on Markov Chain Monte Carlo (MCMC) methods.

## Appendix B. Multicollinearity Diagnostics

### Appendix B.1. Introduction

Multicollinearity occurs when predictor variables in a model are highly correlated, potentially distorting estimates and reducing the interpretability of interaction effects. To assess the multicollinearity in the interaction models used in this study (smoking status × exacerbations × time since diagnosis), diagnostics were conducted.

### Appendix B.2. Methods

1. **Variance Inflation Factor (VIF):**
  o A VIF threshold of 5 was employed to identify potential multicollinearity issues.
  o Each predictor variable and interaction term was evaluated for its VIF.
2. **Condition Number and Variance Proportions:**
  o The condition number, derived from the eigenvalues of the predictor correlation matrix, was calculated to identify multicollinearity.
  o Variance proportions were examined to determine whether any predictors contributed disproportionately to the variance of the smallest eigenvalues.

### Appendix B.3. Results

**Variance Inflation Factor (VIF):**

**Predictor**	**VIF**
Smoking Status	2.2
Exacerbations (≥2/year)	3.0
Time Since Diagnosis (≥5 years)	2.5
Smoking × Exacerbations	3.8
Smoking × Time Since Diagnosis	3.6
Exacerbations × Time Since Diagnosis	4.2
Smoking × Exacerbations × Time Since Diagnosis	4.5

**Condition Number:**

**Statistic**	**Value**
Largest Eigenvalue	12.5
Smallest Eigenvalue	0.25
Condition Number	22.4

**Variance Proportions:**

**Predictor**	**Smallest Eigenvalue Variance Proportion**
Smoking Status	0.15
Exacerbations (≥2/year)	0.12
Time Since Diagnosis (≥5 years)	0.10
Smoking × Exacerbations	0.11
Smoking × Time Since Diagnosis	0.13
Exacerbations × Time Since Diagnosis	0.14
Smoking × Exacerbations × Time Since Diagnosis	0.25

### Appendix B.4. Discussion

The multicollinearity diagnostics, including VIF, condition number, and variance proportions, demonstrate that the ANCOVA models used in this study are robust, with no significant multicollinearity concerns. This ensures reliable estimation of the interaction effects (smoking status × exacerbations × time since diagnosis) on densitometric progression (PD-15 loss and HU-950 change).

### Appendix B.5. Conclusions

Multicollinearity was not a concern in the models employed in this study. This strengthens the validity of the reported interaction effects and their interpretation.

## References

[1] Newnham M., Quinn M., Turner A.M. (2023). Estimating the Prevalence of AATD Patients in the UK to Identify Underdiagnosis and Determine the Eligibility for Potential Augmentation Therapy. Int. J. Chronic Obstr. Pulm. Dis..

[2] Sandhaus R.A., Turino G., Brantly M.L., Campos M., Cross C.E., Goodman K., Hogarth D.K., Knight S.L., Stocks J.M., Stoller J.K. (2016). The Diagnosis and Management of Alpha-1 Antitrypsin Deficiency in the Adult. Chronic Obstr. Pulm. Dis..

[3] Abboud R.T., Nelson T.N. (2006). Alpha1-Antitrypsin Deficiency: A Genetic Risk Factor for COPD. Am. J. Med. Sci..

[4] Silverman E.K., Sandhaus R.A. (2009). Clinical Practice: Alpha1-Antitrypsin Deficiency. N. Engl. J. Med..

[5] Brode S.K., Ling S.C., Chapman K.R. (2012). Alpha-1 Antitrypsin Deficiency: A Commonly Overlooked Cause of Lung Disease. Can. Med Assoc. J..

[6] Stoller J.K., Aboussouan L.S. (2012). A Review of α1-Antitrypsin Deficiency. Am. J. Respir. Crit. Care Med..

[7] Miravitlles M., Dirksen A., Ferrarotti I., Koblizek V., Lange P., Mahadeva R., McElvaney N.G., Parr D., Piitulainen E., Roche N. (2017). European Respiratory Society Statement: Diagnosis and Treatment of Pulmonary Disease in α1-Antitrypsin Deficiency. Eur. Respir. J..

[8] Blanco I., Diego I., Castañón C., Bueno P., Miravitlles M. (2023). Estimated Worldwide Prevalence of the PI*ZZ Alpha-1 Antitrypsin Genotype in Subjects with Chronic Obstructive Pulmonary Disease. Arch. Bronconeumol..

[9] Gadek J.E., Klein H.G., Holland P.V., Crystal R.G. (1981). Replacement therapy of alpha 1-antitrypsin deficiency. Reversal of protease-antiprotease imbalance within the alveolar structures of PiZ subjects. J. Clin. Investig..

[10] Carroll T.P., O’Connor C.A., Floyd O., Lynam P., McElvaney N.G. (2011). The Prevalence of Alpha-1 Antitrypsin Deficiency in Ireland. Respir. Res..

[11] Choate R., Mannino D.M., Holm K.E., Sandhaus R.A. (2019). Comparing patients with ZZ versus SZ alpha-1 antitrypsin deficiency: Findings from AlphaNet’s disease management program. Chronic Obstr. Pulm. Dis..

[12] McElvaney G.N., Sandhaus R.A., Miravitlles M., Turino G.M., Seersholm N., Wencker M., Stockley R.A. (2020). Clinical considerations in individuals with α_1_-antitrypsin PI*SZ genotype. Eur. Respir. J..

[13] Miravitlles M., Turner A.M., Torres-Duran M., Tanash H., Rodríguez-García C., López-Campos J.L., Chlumsky J., Guimaraes C., Rodríguez-Hermosa J.L., Corsico A. (2022). Clinical and functional characteristics of individuals with alpha-1 antitrypsin deficiency: EARCO international registry. Respir. Res..

[14] Greulich T., Albert A., Cassel W., Boeselt T., Peychev E., Klemmer A., Ferreira F.P., Clarenbach C., Torres-Duran M.L., Turner A.M. (2022). Opinions and Attitudes of Pulmonologists About Augmentation Therapy in Patients with Alpha-1 Antitrypsin Deficiency. Int. J. Chronic Obstr. Pulm. Dis..

[15] Dahl M., Hersh C.P., Ly N.P., Berkey C.S., Silverman E.K., Nordestgaard B.G. (2005). The Protease Inhibitor PI*S Allele and COPD: A Meta-Analysis. Eur. Respir. J..

[16] Green C.E., Vayalapra S., Hampson J.A., Mukherjee D., Stockley R.A., Turner A.M. (2015). PiSZ Alpha-1 Antitrypsin Deficiency (AATD): Pulmonary Phenotype and Prognosis Relative to PiZZ AATD and PiMM COPD. Thorax.

[17] Stockley R.A., Edgar R.G., Strange C., Turner A.M. (2015). Antitrypsin Deficiency Assessment and Programme for Treatment (ADAPT): The United Kingdom Registry. J. Chronic Obstr. Pulm. Dis..

[18] Piitulainen F., Tornling G., Eriksson S., Lu Z., Freeman G.L., McCormack J. (1997). Effect of Age and Occupational Exposure to Airway Irritants on Lung Function in Non-Smoking Individuals with Alpha-1 Antitrypsin Deficiency (PiZZ). Thorax.

[19] Stockley R.A., Edgar R.G., Pillai A., Turner A.M. (2016). Individualized Lung Function Trends in Alpha-1 Antitrypsin Deficiency: A Need for Patience to Provide Patient-Centered Management. Int. J. Chronic Obstr. Pulm. Dis..

[20] Tanash H.A., Nilsson P.M., Nilsson J.A., Piitulainen E., Lu Z. (2010). Survival in Severe Alpha-1 Antitrypsin Deficiency (PiZZ). Respir. Res..

[21] McElvaney N.G., Stoller J.K., Buist A.S., Prakash U.B., Brantly M.L., Schluchter M.D., Crystal R.D. (1997). Baseline Characteristics of Enrollees in the National Heart, Lung, and Blood Institute Registry of Alpha 1-Antitrypsin Deficiency. Chest.

[22] Esquinas C., Serreri S., Barrecheguren M., Rodriguez E., Nuñez A., Casas-Maldonado F., Blanco I., Pirina P., Lara B., Miravitlles M. (2018). Long-Term Evolution of Lung Function in Individuals with Alpha-1 Antitrypsin Deficiency from the Spanish Registry (REDAAT). Int. J. Chronic Obstr. Pulm. Dis..

[23] Holme J., Stockley R.A. (2007). Radiologic and Clinical Features of COPD Patients with Discordant Pulmonary Physiology: Lessons from Alpha-1 Antitrypsin Deficiency. Chest.

[24] Ward H., Turner A.M., Stockley R.A., McElvaney N.G. (2014). Spirometric and Gas Transfer Discordance in Alpha-1 Antitrypsin Deficiency: Patient Characteristics and Progression. Chest.

[25] McElvaney N.G., Burdon J., Holmes M., Glanville A., Wark P.A., Thompson P.J., Hernandez P., Chlumsky J., Teschler H., Ficker J.H. (2017). Long-Term Efficacy and Safety of α1 Proteinase Inhibitor Treatment for Emphysema Caused by Severe α1 Antitrypsin Deficiency: An Open-Label Extension Trial (RAPID-OLE). Lancet Respir. Med..

[26] Stockley R.A., Parr D.G., Piitulainen E., Stolk J., Stoel B.C., Dirksen A. (2010). Therapeutic Efficacy of α1-Antitrypsin Augmentation Therapy on the Loss of Lung Tissue: An Integrated Analysis of 2 Randomized Clinical Trials Using Computed Tomography Densitometry. Respir. Res..

[27] Chapman K.R., Burdon J.G., Piitulainen E., Sandhaus R.A., Seersholm N., Stocks J.M., Stoel B.C., Huang L., Yao Z., Edelman J.M. (2015). Intravenous Augmentation Treatment and Lung Density in Severe α1 Antitrypsin Deficiency (RAPID): A Randomised, Double-Blind, Placebo-Controlled Trial. Lancet.

[28] Belmonte I., Barrecheguren M., Esquinas C., Rodriguez E., Miravitlles M., Rodríguez-Frías F. (2017). Genetic Diagnosis of Alpha1-Antitrypsin Deficiency Using DNA from Buccal Swab and Serum Samples. Clin. Chem. Lab. Med..

[29] American Thoracic Society Statement (1991). Lung Function Testing: Selection of Reference Values and Interpretative Strategies. Am. Rev. Respir. Dis..

[30] Macintyre N., Crapo R.O., Viegi G., Johnson D.C., van der Grinten C.P., Brusasco V., Burgos F., Casaburi R., Coates A., Enright P. (2005). Standardisation of the Single-Breath Determination of Carbon Monoxide Uptake in the Lung. Eur. Respir. J..

[31] Quanjer P.H., Stanojevic S., Cole T.J., Baur X., Hall G.L., Culver B.H., Enright P.L., Hankinson J.L., Ip M.S.M., Zheng J. (2012). Multi-Ethnic Reference Values for Spirometry for the 3–95-Yr Age Range: The Global Lung Function 2012 Equations. Eur. Respir. J..

[32] Baraghoshi D., Strand M., Humphries S.M., San José Estépar R., Vegas Sanchez-Ferrero G., Charbonnier J.P., Latisenko R., Silverman E.K., Crapo J.D., Lynch D.A. (2023). Quantitative CT Evaluation of Emphysema Progression over 10 Years in the COPDGene Study. Radiology.

[33] Holme J., Stockley J.A., Stockley R.A., Jehpsson L., Tanash H.A. (2013). Age-Related Development of Respiratory Abnormalities in Non-Index α1-Antitrypsin Deficient Studies. Respir. Med..

[34] Dowson L.J., Guest P.J., Stockley R.A. (2001). Longitudinal Changes in Physiological, Radiological, and Health Status Measurements in Alpha(1)-Antitrypsin Deficiency and Factors Associated with Decline. Am. J. Respir. Crit. Care Med..

[35] Crossley D., Renton M., Khan M., Low E.V., Turner A.M. (2018). CT Densitometry in Emphysema: A Systematic Review of Its Clinical Utility. Int. J. Chronic Obstr. Pulm. Dis..

[36] Parr D.G., Stoel B.C., Stolk J., Stockley R.A. (2006). Validation of Computed Tomographic Lung Densitometry for Monitoring Emphysema in Alpha1-Antitrypsin Deficiency. Thorax.

[37] Tejwani V., Nowacki A.S., Fye E., Sanders C., Stoller J.K. (2019). The Impact of Delayed Diagnosis of Alpha-1 Antitrypsin Deficiency: The Association Between Diagnostic Delay and Worsened Clinical Status. Respir. Care.

[38] Turino G.M., Barker A.F., Brantly M.L., Cohen A.B., Connelly R.P., Schluchter M.D., Crystal R.G., Eden E., Stoller J.K. (1996). Clinical Features of Individuals with PI*SZ Phenotype of Alpha 1-Antitrypsin Deficiency. Am. J. Respir. Crit. Care Med..

[39] Franciosi A.N., Hobbs B.D., McElvaney O.J., Molloy K., Hersh C.P., Clarke L., Gunaratnam C., Silverman E.K., Carroll T.P., McElvaney N.G. (2020). Clarifying the Risk of Lung Disease in SZ Alpha-1 Antitrypsin Deficiency. Am. J. Respir. Crit. Care Med..

[40] Crossley D., Stockley J., Bolton C.E., Hopkinson N.S., Mahadeva R., Steiner M., Wilkinson T., Hurst J.R., Gooptu B., Stockley R.A. (2020). Relationship of CT densitometry to lung physiological parameters and health status in alpha-1 antitrypsin deficiency: Initial report of a centralised database of the NIHR rare diseases translational research collaborative. BMJ Open.

[41] Fähndrich S., Bernhard N., Lepper P.M., Vogelmeier C., Seibert M., Wagenpfeil S., Bals R. (2017). Exacerbations and Duration of Smoking Abstinence Are Associated with the Annual Loss of FEV1 in Individuals with PiZZ Alpha-1 Antitrypsin Deficiency. Respir. Med..

[42] Hiller A.M., Piitulainen E., Jehpsson L., Tanash H.A. (2019). Decline in FEV1 and Hospitalized Exacerbations in Individuals with Severe Alpha-1 Antitrypsin Deficiency. Int. J. Chronic Obstr. Pulm. Dis..

[43] Omachi T.A., Eisner M.D., Rames A., Markovtsova L., Blanc P.D. (2011). Matrix Metalloproteinase-9 Predicts Pulmonary Status Declines in α1-Antitrypsin Deficiency. Respir. Res..

[44] Pierce L.R. (2024). Assessing the efficacy of Alpha_1_-Proteinase inhibitor (human) augmentation therapy for Alpha_1_-Antitrypsin deficiency-Related emphysema: Challenges and opportunities. Heliyon.

[45] Ostridge K., Wilkinson T.M. (2016). Present and Future Utility of Computed Tomography Scanning in the Assessment and Management of COPD. Eur. Respir. J..

[46] Stoel B.C., Stolk J., Bakker M.E., Parr D.G. (2019). Regional Lung Densities in Alpha-1 Antitrypsin Deficiency Compared to Predicted Values. Respir. Res..

