# Supplementary material for: Densitometric and Functional Progression in Patients with Alpha-1 Antitrypsin Deficiency Genotype SZ
Source: J Clin Med. 2025 Mar 4;14(5):1725. doi: 10.3390/jcm14051725 (PMC11900030; doi:10.3390/jcm14051725)
Supplement: Supplementary file 1 [file jcm-14-01725-s001.zip › Supplementary Figure S1.pdf]

Supplementary

Figure S1: Correlations between densitometric parameters (PD-15 and HU-950) and functional parameters (FEV1, DLCO, KCO) according to time since AATD diagnosis (<5 years and ≥5 years).

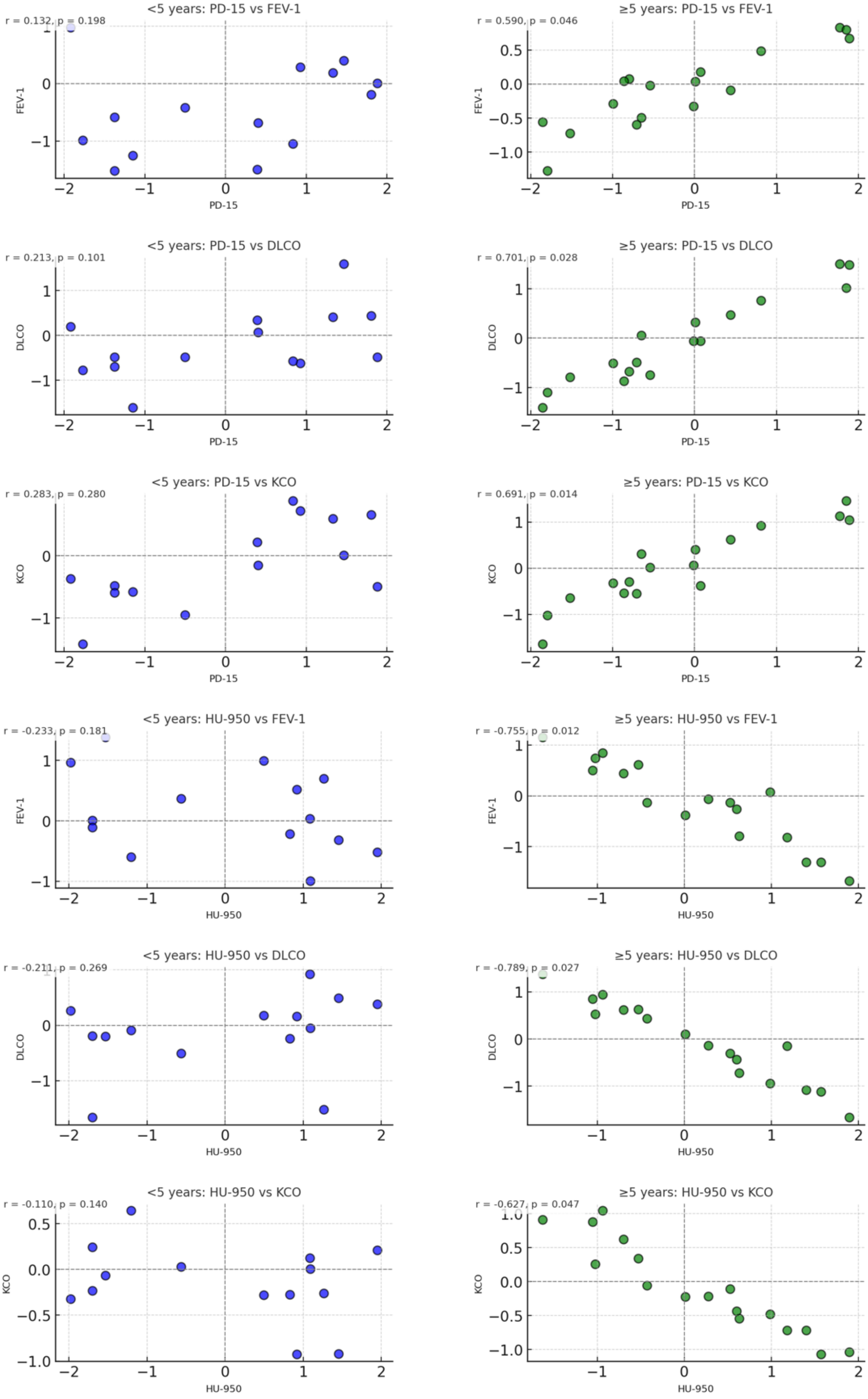

Footnote: PD-15: 15th percentile lung density; HU-950: lung volume with density less than -950 HU; FEV1: forced expiratory volume in 1 second; DLCO: carbon monoxide diffusion capacity; KCO: carbon monoxide transfer coefficient.
