# Supplementary material for: Densitometric and Functional Progression in Patients with Alpha-1 Antitrypsin Deficiency Genotype SZ
Source: J Clin Med. 2025 Mar 4;14(5):1725. doi: 10.3390/jcm14051725 (PMC11900030; doi:10.3390/jcm14051725)
Supplement: Supplementary file 1 [file jcm-14-01725-s001.zip › Supplementary Figure S2.pdf]

Figure S2: Interaction effects of smoking status, exacerbation frequency, and time since diagnosis on densitometric progression in PiSZ patients**.** Annual decline in PD-15(a), Annual increase in HU-950 (b).

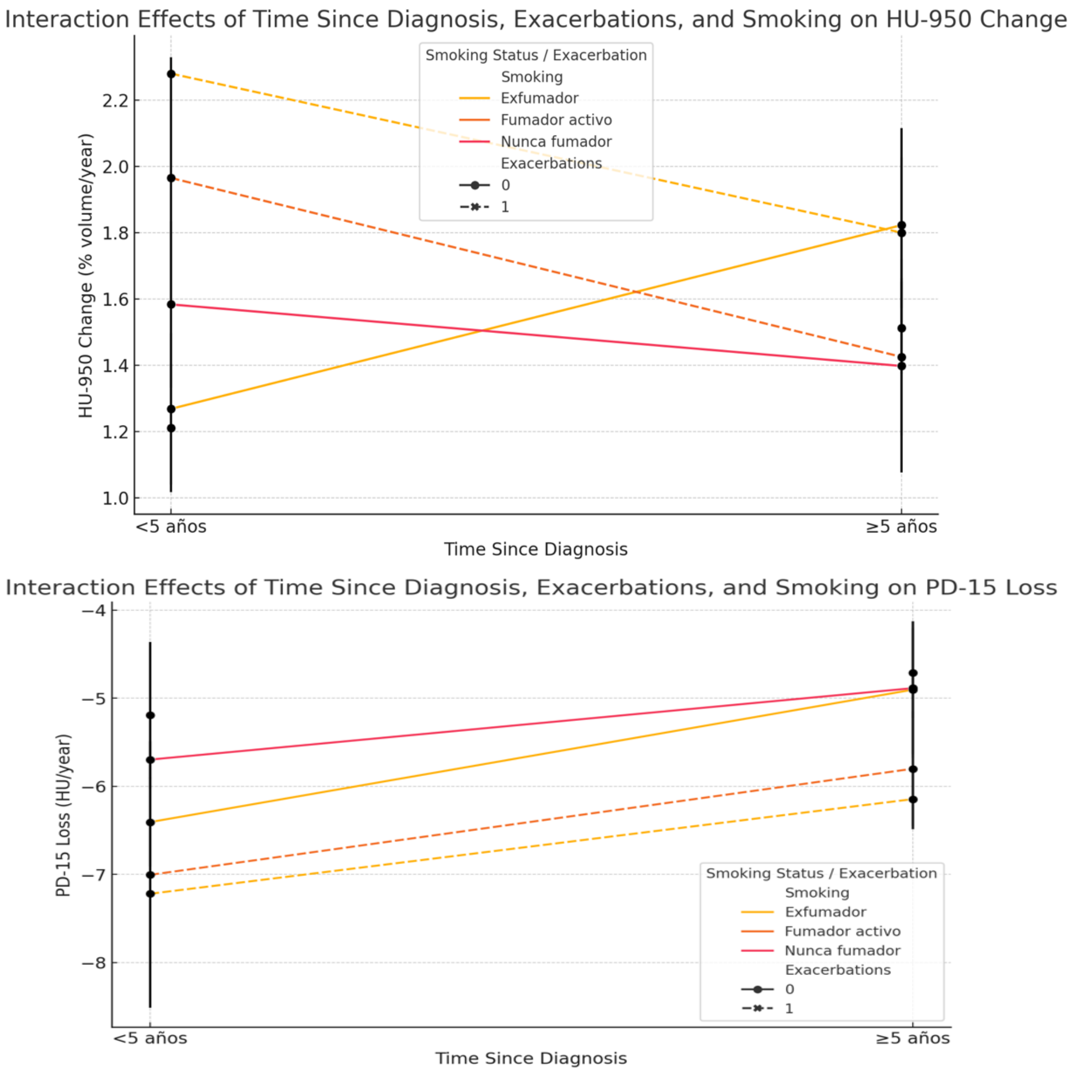

Footnote: The solid lines represent patients without frequent exacerbations (<2/year, labeled as "0"), while the dashed lines represent those with frequent exacerbations (≥2/year, labeled as "1"). The interaction between smoking status, time since diagnosis, and exacerbation frequency is shown for each subgroup, illustrating their combined impact on PD-15 loss (Figure 2a) and HU-950 change (Figure 2b). PD-15: 15th percentile lung density; HU-950: lung volume with density less than -950 HU.
